# Preparation of Heterojunctions Based on Cs_3_Bi_2_Br_9_ Nanocrystals and g-C_3_N_4_ Nanosheets for Photocatalytic Hydrogen Evolution

**DOI:** 10.3390/nano13020263

**Published:** 2023-01-07

**Authors:** María Medina-Llamas, Andrea Speltini, Antonella Profumo, Francesca Panzarea, Antonella Milella, Francesco Fracassi, Andrea Listorti, Lorenzo Malavasi

**Affiliations:** 1 Unidad Académica Preparatoria, Plantel II, Universidad Autónoma de Zacatecas, Zacatecas 98068, Mexico; 2 Department of Chemistry, University of Pavia, Via Taramelli 12, 27100 Pavia, Italy; 3 Department of Chemistry, University of Bari “Aldo Moro” via Orabona 4, 70126 Bari, Italy; 4 National Research Council, Department of Chemistry, Institute of Nanotechnology (CNR-NANOTEC), 70125 Bari, Italy

**Keywords:** lead-free perovskites, hydrogen evolution, photocatalysis, carbon nitride, perovskites, ball milling

## Abstract

Heterojunctions based on metal halide perovskites (MHPs) are promising systems for the photocatalytic hydrogen evolution reaction (HER). In this work, we coupled Cs_3_Bi_2_Br_9_ nanocrystals (NCs), obtained by wet ball milling synthesis, with g-C_3_N_4_ nanosheets (NSs), produced by thermal oxidation of bulk g-C_3_N_4,_ in air. These methods are reproducible, inexpensive and easy to scale up. Heterojunctions with different loadings of Cs_3_Bi_2_Br_9_ NCs were fully characterised and tested for the HER. A relevant improvement of H_2_ production with respect to pristine carbon nitride was achieved at low NCs levels reaching values up to about 4600 µmol g^−1^ h^−1^. This work aims to provide insights into the synthesis of inexpensive and high-performing heterojunctions using MHP for photocatalytic applications.

## 1. Introduction

In recent times, the field of application of metal halide perovskites (MHPs) has been expanded to photocatalysis, thanks to their excellent optical properties such as high optical absorption coefficient, high carrier mobility and long electron-hole diffusion lengths [1]. In this vast area of applications, ranging from hydrogen photogeneration and carbon dioxide reduction to H_2_O_2_ production to organic dye degradation [2,3,4,5,6]; the need for using lead-free materials is a key aspect of considering the final aims of such uses of MHPs. In general, lead is replaced by elements with similar electronic structure and/or comparable ionic radius, such as Sn^2+^, Ge^2+^, Sb^3+^ or Bi^3+^. To date, several examples of the use of lead-free MHPs in photocatalysis have been reported [7,8]. Among these results, a class of interesting phases, showing good photocatalytic performances, is that of Cs_3_Bi_2_X_9_ perovskites derivatives which display high stability in water, light and heat, combined with their low toxicity and the relatively low cost of bismuth [9]. By way of example, Bresolin et al., (2020) produced bulk Cs_3_Bi_2_I_9_ through a precipitation method and tested it for the photocatalytic degradation of dyes [10]. The same application was explored by Akinbami et al., (2021) by using Cs_3_Bi_2_Br_9_ nanocrystals (NCs) prepared using a hot injection method, while [11] Bhosale et al., (2019) synthetised Cs_3_Bi_2_Br_9_ NCs via ultrasonication for the photoreduction of carbon monoxide [12]. In general, a further enhancement of the MHPs photocatalytic activity is achieved by using them in combination with a second semiconductor, as is commonly performed in current research, i.e., by creating heterojunctions. Among other visible-light active catalysts, graphitic carbon nitride (g-C_3_N_4_) has been already advantageously used to create composite systems with lead-free MHPs [13,14].

g-C_3_N_4_, is a 2D layered material that has rapidly emerged as a promising photocatalyst. It can be easily synthesised from the thermal polycondensation of inexpensive precursors that contain nitrogen and carbon atoms such as dicyanamide, cyanamide, melamine, urea and thiourea. Its suitable bandgap, ~2.8 eV, makes it active in the visible solar spectrum [15]. It is a thermally and chemically stable compound, resistant to acid or alkaline conditions. On the other hand, g-C_3_N_4_ has a high recombination rate of its electron-hole pairs and a low surface area. The first drawback is due to the presence of defects as a result of incomplete deamination during the thermal condensation process [16]. These defects act as recombination centres, leading to a decrease in the photocatalytic activity of the material. The low surface area of g-C_3_N_4_ can be overcome by exfoliation methods such as thermal, ultrasonic or chemical exfoliation [17,18,19]. Thanks to these strategies, it is possible to obtain g-C_3_N_4_ nanosheets (g-C_3_N_4_ NSs) that have a high aspect-ratio, thin thickness and plenty of surface groups for the anchoring of co-catalysts [20]. Consequently, exfoliation methods are widely used to increase the surface area of bulk g-C_3_N_4_. Among them, ultrasonication is an energy and time-consuming process that usually does not produce single layers of g-C_3_N_4_ [20]. Chemical exfoliation is achieved using strong acids (H_2_SO_4_ and/or HNO_3_) but is time-consuming [18]. By contrast, thermal exfoliation is a low-cost, easy scale-up and environmentally-friendly method to obtain single layers.

Our research group recently explored the use of bulk g-C_3_N_4_ in the construction of heterojunctions with microcrystalline Cs_3_Bi_2_Br_9_ for hydrogen production, achieving a hydrogen evolution rate (HER) up to about 1050 µmol g^−1^ h^−1^ compared to 81 µmol g^−1^ h^−1^ of bulk g-C_3_N_4_ [21]. Based on these results, we decided to further investigate this heterojunction to boost the production of hydrogen through the combination of nanocrystalline Cs_3_Bi_2_Br_9_ and g-C_3_N_4_ NSs. The aim is to obtain nanostructured composites that can be easily manufactured at a large scale, using simple preparation routes. For this purpose, the Cs_3_Bi_2_Br_9_ NCs were synthesised by wet ball milling instead of using standard liquid-phase strategies that require a high temperature (130–220 °C), inert gas atmosphere and the use of large quantities of organic solvents (i.e., hot injection or ligand-assisted reprecipitation) [22]. Indeed, these methods produce small quantities of NCs that need further purification, making the whole process expensive, energy consuming and not in line with green chemistry principles. To overcome the former disadvantages, a mechano-chemical procedure using a planetary ball miller was chosen in this work. Through this method, the synthesis of perovskites takes place primarily via high-energy impacts between the grinding balls and the grinding bowl (which contains the perovskite precursors). The grinding balls move across the bowl at high speed and hit the perovskite precursors, resulting in mechanical downsizing and the chemical reaction of the perovskite precursors [23]. Ball milling synthesis can be conducted without solvents (dry synthesis) and with solvents (wet synthesis). Both approaches are reproducible and scalable. Wet ball milling (WBM) is a green chemistry synthesis method and does not require an inert atmosphere or high temperature [24]. The experiments conducted in this research were carried out using a laboratory-scale ball mill in a batch mode, unlike industrial ball mills which operate in a continuous mode [25], thus a high synthesis throughput can be easily achieved.

Based on the previous considerations, the strategy used in the present work to overcome the limitations of bulk g-C_3_N_4_ is based on increasing its surface area through a thermal exfoliation process followed by the construction of a heterojunction with Cs_3_Bi_2_Br_9_ NCs via wet ball milling. As already demonstrated for a similar bulk heterojunction [21], there is a favourable band-alignment between the two semiconductors that promotes charge transfer and reduces the charge recombination rate of g-C_3_N_4_. In addition to this, the nanostructured morphology of the present heterojunction is expected to provide a more efficient route for the photocatalytic activity of the Cs_3_Bi_2_Br_9_ NCs/g-C_3_N_4_ NSs composites.

## 2. Materials and Methods

### 2.1. Synthesis of Bulk g-C_3_N_4_ and g-C_3_N_4_ Nanosheets

Bulk g-C_3_N_4_ was synthetised by the polymerisation of dicyandiamide (DCD, 99%, Aldrich, Italy) using a thermal treatment under N_2_ atmosphere. In the process, an alumina crucible was completely filled with DCD and heated up to 550 °C with a ramp of 1 °C min^−1^ and a dwell time of 4 h, followed by a cooling step to room temperature. Later, the bulk g-C_3_N_4_ was finely ground with a mortar and a pestle. The g-C_3_N_4_ NSs were obtained by subjecting the bulk g-C_3_N_4_ to a second calcination in air. The calcination of 500 mg of bulk g-C_3_N_4_ at 500 °C with a ramp of 5 °C min^−1^ and 2 h of dwell was performed, as reported in a previous publication [18].

### 2.2. Synthesis of Bulk Cs_3_Bi_2_Br_9_ and Cs_3_Bi_2_Br_9_ NCs

The Cs_3_Bi_2_Br_9_ bulk perovskite was prepared by a solid-state synthesis using a planetary milling (pulverisette 7, Fritsch,, Milan, Italy). In total, 1.5 g of Cs_3_Bi_2_Br_9_ bulk perovskite was prepared by adding the stochiometric quantities of caesium bromide (CsBr 99.9%, Sigma Aldrich, Italy) and bismuth bromide III (BiBr_3_ 99%, Sigma Aldrich, Italy) in each tungsten carbide grinding bowl. Then, fifteen tungsten carbide balls (D = 5 mm) were added into each grinding bowl to achieve a ratio of 10 balls/g _solids_. The milling was performed in 3 cycles of 20 min at 500 rpm with a 10 min pause between each cycle. Cs_3_Bi_2_Br_9_ NCs were produced by a LARP (ligand-assisted reprecipitation) method using a planetary milling. For the synthesis, 0.07 mmol of the previously synthesised Cs_3_Bi_2_Br_9_ bulk perovskite, 0.1 mL oleic acid (OA 99%, Sigma Aldrich, Italy), 0.1 mL oleylamine (OLA 98%, Sigma Aldrich, Italy) and 0.8 mL toluene (99.5%, Sigma Aldrich, Italy) were added in a tungsten carbide bowl. For this step, a ratio of 40 balls/g _solids_ was selected. The milling was carried out with 2 cycles of 15 min at 500 rpm with a 10 min pause between the cycles. The synthesis of the Cs_3_Bi_2_Br_9_ NCs was easily scaled up by adding more reagents while keeping the ratio of 40 balls/g _solids_. The obtained suspension was retrieved from the grinding bowls and subjected to centrifugation at 8 500 rpm for 10 min to remove the excess of ligands. The supernatant was discarded and the NCs were redispersed in fresh toluene. This step was repeated two more times. Next, the NCs suspension was subjected to a mild centrifugation at 3 000 rpm to remove large aggregates. Finally, the supernatant containing the Cs_3_Bi_2_Br_9_ NCs was carefully retrieved. 

### 2.3. Synthesis of Cs_3_Bi_2_Br_9_ NCs/g-C_3_N_4_ Nanosheets

The composites between Cs_3_Bi_2_Br_9_ NCs and g-C_3_N_4_ NSs were produced via a wet chemistry step by mixing the Cs_3_Bi_2_Br_9_ NCs suspended in toluene with g-C_3_N_4_ NSs, previously sonicated for 10 min in fresh toluene. The system was kept under agitation at 500 rpm and heated up to 50 °C until complete evaporation of the solvent was achieved. Nominal amounts of NCs loading on g-C_3_N_4_ NSs were 0.05, 0.5, 1 and 1.5%. The effective loading was determined by acidic dissolution measured by quantitation of the bismuth measured by inductively coupled plasma-optical emission spectroscopy (ICP-OES), as described below.

### 2.4. Characterisation 

The crystal structure of the samples was acquired at room temperature Cu-radiation XRD using a Bruker D2 diffractometer (Bremen, Germany). Diffuse reflectance spectroscopy spectra were obtained in the wavelength range of 250–850 nm using a Jasco V-750 spectrophotometer (Cremella, Italy), equipped with an integrating sphere (Jasco ISV-922). Microstructural characterisation of the samples was achieved via a high-resolution scanning electron microscope (SEM, TESCAN Mira 3, Czech Republic) operated at 20 kV. Surface area measures were carried out via Brunauer, Emmett and Teller (BET) single-point method using a Flowsorb II 2300 (Micromeritics, United States) apparatus. Each sample was accurately weighed (0.3 g) and degassed at 80 °C for 15 h under a continuous stream of N_2_:He 30:70 mixture. Gas adsorption was achieved by placing the sample in liquid nitrogen. TEM micrographs of Cs_3_Bi_2_Br_9_ NCs were obtained by means of a JEOL JEM-1200 EX II (Milan, Italy) microscope operating at 100 kV, equipped with a tungsten filament as the electron source.

Quantification of the amount of perovskite in the composites was achieved by determination of bismuth by ICP-OES (Perkin Elmer Optima 3300 DV, Italy) after complete dissolution of the perovskite fraction. In detail, 10 mg of each composite and 2 mL of concentrated HNO_3_ (69%, Fisher, ICP-OES, for Trace Metal Analysis, Italy) were placed in glass beakers. The suspension was kept under agitation for 3 h, under reflux conditions. Later, the beakers were left open, the temperature was slightly increased to evaporate most of the acid. Subsequently, 8 mL of tri-distilled water was carefully added to each glass vessel to decrease the acidity of the suspension. Finally, the suspension was filtrated on a 0.45 µm nylon membrane (syringe filter unit, Whatman, Sigma Aldrich, Italy) and diluted to a final volume of 10 mL with distilled water. The entire process was conducted in the fume hood. The weight percentage of the perovskite in the composites was determined by the quantification of bismuth in the obtained solutions by external calibration; Bi standard solutions were prepared in distilled water 1% *v/v* ultrapure HNO_3_, in the concentration range 1–50 mg L^−1^. The photoluminescence (PL) measurements were recorded by means of a Fluorolog^®^-3 spectrofluorometer (HORIBA Jobin-Yvon, Roma, Italy), equipped with a 450 W xenon lamp as an exciting source and double grating excitation and emission monochromators. All the optical measurements were performed at room temperature on powder-dispersed samples as obtained from the synthesis without any size sorting treatment. The PL emission spectra were recorded by using an excitation wavelength of 350 nm. Time-Resolved PL (TRPL) measurements were carried out by Time Correlated Single Photon Counting (TCSPC) technique, with a FluoroHub (HORIBA Jobin-Yvon). CDs solutions were excited using 80 ps laser diode sources at 375 nm (NanoLED 375L). The time resolution was ~300 ps for all the measurements.

Surface chemical composition was investigated by XPS analyses with a PHI 5000 Versa Probe II spectrometer (Physical Electronics, Roma, Italy) equipped with a monochromatic Al Kα X-ray source (1486.6 eV), operated at 15 kV and 24.8 W, with a spot size of 100 µm. Survey (0–1400 eV) and high-resolution spectra (C1s, O1s, N1s, Br3d Cs3d and Bi4f) were recorded in FAT (Fixed Analyser Transmission) mode at a pass energy of 117.40 and 29.35 eV, respectively. Surface charging was compensated using a dual beam charge neutralisation system. The hydrocarbon component of the C1s spectrum was used as the internal standard for charging correction and it was fixed at 284.8 eV. Spectra were processed with MultiPak software (Physical Electronics).

### 2.5. Photocatalytic Hydrogen Experiments

Hydrogen evolution experiments took place in Pyrex glass containers (32 mL) containing 24 mL of a 10% *v/v* triethanolamine aqueous solution (TEOA ≥ 99%), SigmaAldrich, Italyand 24 mg of the photocatalyst (1 g _catalyst_/L _solution_). Oxygen was removed by argon bubbling for 20 min. Prior to sealing the glass containers with sleeve stopper septum, platinum was added as a co-catalyst, by pouring 40 µL of a 0.08 M H_2_PtCl_6_ solution (chloroplatinic acid hydrate, 38% Pt basis, Sigma Aldrich, Italy). During the irradiation, platinum is photoreduced and, in situ photodeposited on the catalyst surface. Irradiation was performed under simulated solar light (1500 W Xenon lamp, 300–800 nm, IR-treated soda lime glass UV outdoor filter) at 500 W m^−2^ for 6 h under magnetic stirring, using a Solar Box 1500e (COFOMEGRA Srl, Milan, Italy). Triplicate experiments were performed on all samples. The headspace-evolved gas was quantified by gas chromatography coupled with thermal conductivity detection (GC-TCD, Dani SPA, Italy). The HER is expressed as μmol per gram of catalyst per hour of irradiation (µmol g^−1^ h ^−1^).

## 3. Results and Discussion

A set of composites consisting of Cs_3_Bi_2_Br_9_ NCs and g-C_3_N_4_ NSs with different perovskite loadings were prepared according to the procedure described in the Experimental section. The effective weight percentage of perovskite in each composite was calculated through the Bi content measured by ICP-OES (as described in Section 2.4). The percentages of perovskite loading, in terms of per cent weight, in the final composites 0.02, 0.44, 0.91 and 1.47% were in good agreement with nominal starting values of 0.05, 0.5, 1 and 1.5%. The composites were extensively characterised in terms of crystal structure, optical properties and morphology. 

**Figure 1:**
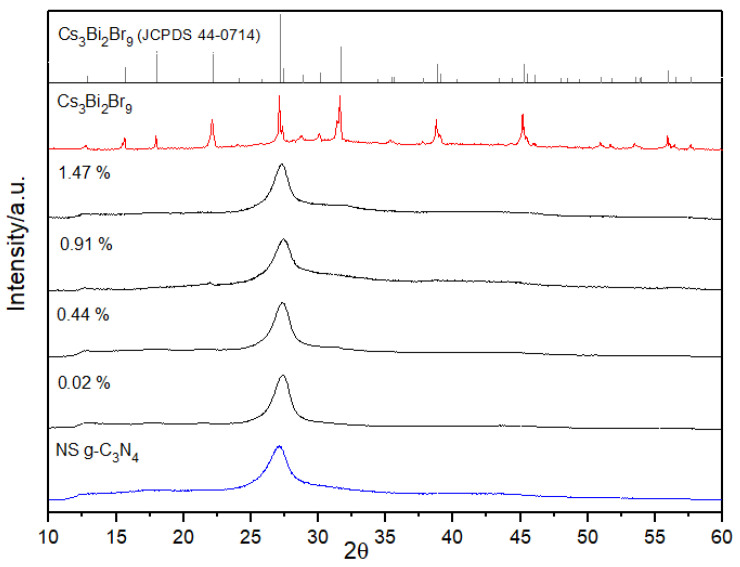
X-ray diffraction patterns of the g-C_3_N_4_ nanosheets, the Cs_3_Bi_2_Br_9_ nanocrystals, Cs_3_Bi_2_Br_9_ NCs/g-C_3_N_4_ NSs composites produced at different percentages of perovskite loading (wt.%) and the reference pattern of the Cs_3_Bi_2_Br_9_.

Figure 1 shows the X-ray diffraction patterns of all prepared materials, with the g-C_3_N_4_ NSs at the bottom of the figure, followed by the XRD pattern of the composites and the pattern of Cs_3_Bi_2_Br_9_ NCs. At the top of the figure, the reference pattern of the Cs_3_Bi_2_Br_9_ (JCPDS 44-0714) can be found. The experimental g-C_3_N_4_ pattern (JCPDS 87-1526) is characterised by the broad peak at approximately 27° which is clearly visible in all the patterns of the composites, being carbon nitride the main phase. The contribution of the Cs_3_Bi_2_Br_9_ NCs to the patterns can only be slightly appreciated at the two highest loadings (0.91 and 1.47 wt.%) where weak intensities at about 22.0° and 31.6° can be found corresponding to the (102) and (202) planes, respectively. Figure S1 compares the X-ray diffraction pattern of bulk g-C_3_N_4_ and g-C_3_N_4_ NSs. Both samples have the main peaks at 13° and 27° that correspond to the in-plane structural packing motifs (100) and the interlayer stacking of the conjugated aromatic systems (002), respectively [17,26]. Both peaks become broader and less pronounced for the g-C_3_N_4_ NSs, suggesting a decrease of size in both directions (i.e., parallel, and vertical to the C_3_N_4_ layers) caused by the thermal oxidation process and a partial loss of structural order in the planes. 

**Figure 2:**
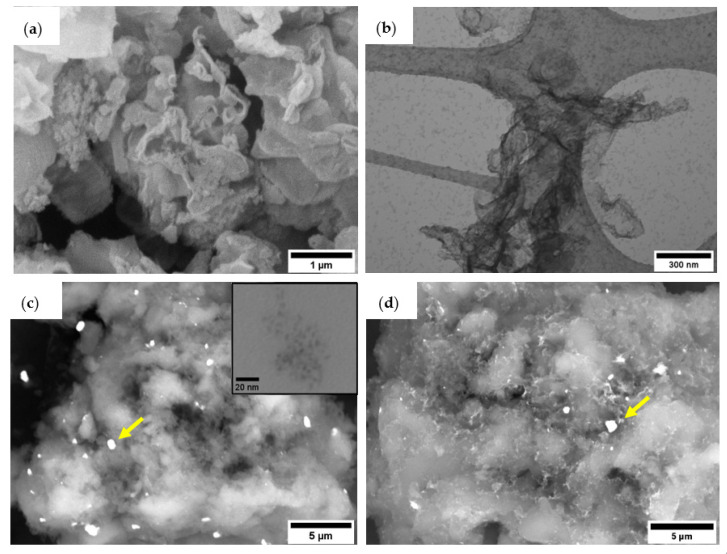
(**a**) SEM and (**b**) TEM micrographs of the g-C_3_N_4_ NSs, BSE-HRSEM of the (**c**) 0.44 wt.%Cs_3_Bi_2_Br_9_ NCs/g-C_3_N_4_ NSs, NCs and (**d**) 1.47 wt.%. Cs_3_Bi_2_Br_9_ NCs/g-C_3_N_4_ NSs. Inset: TEM micrograph of the Cs_3_Bi_2_Br_9_ NCs. The yellow arrows show the selected areas for EDX analysis.

The morphology and microstructure of the g-C_3_N_4_ NSs were investigated by SEM and TEM as shown in Figure 2a,b. Figure 2a confirms that the g-C_3_N_4_ NSs consist of agglomerates of thin sheets that tend to bend at the edges. The TEM micrograph (Figure 2b) demonstrates the formation of nanosheets due to the thermal exfoliation process that weakens the bonds between the layers of bulk g-C_3_N_4_. The yield of the thermal exfoliation process, about 60%, was measured as the ratio between the obtained mass of g-C_3_N_4_ NSs and the initial mass of bulk g-C_3_N_4_. The resulting specific surface area (SSA) values of the g-C_3_N_4_ NSs was 103.5 m^2^ g^−1^, whereas the SSA of the bulk g-C_3_N_4_ was 11.7 m^2^ g^−1^. This is almost a nine-fold increase in the SSA achieved by thermal exfoliation. Figure S2 shows the nitrogen adsorption isotherm of the bulk g-C_3_N_4_ and the g-C_3_N_4_ NSs. For comparison, Figure S3 shows a low magnification SEM micrograph of the bulk g-C_3_N_4,_ showing, for the bulk material, a more compact microstructure. Figure 2c,d show, as selected examples, the backscattered electron (BSE) micrographs of the 0.44% and 1.47 wt.% Cs_3_Bi_2_Br_9_ NCs/g-C_3_N_4_ NSs. In both samples, EDX was conducted to confirm the presence of the Cs_3_Bi_2_Br_9_ NCs in the composites. The yellow arrows in Figure 2c,d show the selected areas for EDX analysis. Figure S4 shows a SEM micrograph of the analysed section, the EDX spectrum and the obtained atomic percentage of each element in the composites. The results confirm the presence of C and N from g-C_3_N_4_ and the presence of Cs, Bi and Br from the Cs_3_Bi_2_Br_9_ NCs. Oxygen was also found, as g-C_3_N_4_ NSs were obtained by a thermal exfoliation process in air. In addition, the inset of Figure 2c shows a TEM image of the starting Cs_3_Bi_2_Br_9_ NCs having an average size of around 4–5 nm. The small particle size is achieved thanks to the mechanical downsizing by WBM and the presence of ligands that prevent the growth of the NCs. Both BSE-HRSEM micrographs show a homogeneous distribution of small Cs_3_Bi_2_Br_9_ NCs over the 2D nanosheets and the presence of a few clusters of Cs_3_Bi_2_Br_9_ NCs of different sizes. Agglomerations of NCs might occur during the preparation of the composite. However, small particles of Cs_3_Bi_2_Br_9_ can be easily seen, especially in Figure 2d. A higher magnification SEM image of 0.44 wt.%. sample is reported in the supporting information (Figure S5) showing in greater detail the homogenous distribution of Cs_3_Bi_2_Br_9_ NCs.

**Figure 3:**
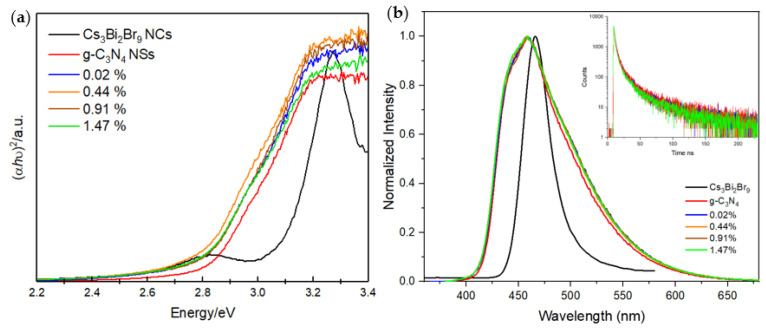
(**a**) Tauc plot and (**b**) normalised emission and time-resolved photoluminescence spectra of the g-C_3_N_4_ NSs, the Cs_3_Bi_2_Br_9_ NCs and the Cs_3_Bi_2_Br_9_ NCs/g-C_3_N_4_ NSs composites (λ_ex_ = 350 nm).

Optical properties of the Cs_3_Bi_2_Br_9_ NCs/g-C_3_N_4_ NSs composites were determined by UV–Vis absorption spectroscopy. Figure 3a shows the Tauc plots for each material, the estimated band gap for the g-C_3_N_4_ NSs was 2.80 eV. The addition of Cs_3_Bi_2_Br_9_ NCs to the g-C_3_N_4_ NSs produced a slight shift towards lower energies with the band gaps moving around 2.77 eV. Figure S6a is a section of the Tauc plot that shows the linear extrapolation to the *x*-axis. Figure S6b shows the absorbance spectra versus wavelength of all samples. Figure 3b shows the normalised photoluminescence spectra of Cs_3_Bi_2_Br_9_ NCs/g-C_3_N_4_ NSs composites measured in the range 360–680 nm, compared with bare g-C_3_N_4_ NSs and Cs_3_Bi_2_Br_9_ NCs spectra. Pure g-C_3_N_4_ NSs shows an emission peak at 459 nm. Compared to it, no shift of the emission peak or change in the peak shape is observed for all the samples, suggesting that a perovskite loading of this entity does not affect the steady-state emission properties characterised by a dominating g-C_3_N_4_ contribution. The time-resolved photoluminescence decay curves of the same samples are shown in the Inset of Figure 3b. It is possible to notice that charge lifetime slightly decreases upon introduction of Cs_3_Bi_2_Br_9_ NCs. By multi-exponential fitting, it can be estimated that the lifetime of bare g-C_3_N_4_ is 12.5 ns, while those of Cs_3_Bi_2_Br_9_ NCs/g-C_3_N_4_ NSs composites are respectively 9.6, 9.1, 8.9 and 8.7 ns with increasing amounts of perovskite loading. These decreasing lifetimes suggest how differences occur in the relative populations of excitation/deactivation processes when increasing amounts of perovskite NCs are loaded on g-C_3_N_4_ NSs. A more efficient charge transfer process does not linearly translate into higher HER, as will be further discussed below, for the presence of self-trapping phenomena occurring in Cs_3_Bi_2_Br_9_ at higher concentrations, we associated this peculiar optical behaviour of perovskite-carbon nitride composites to excitation/deactivation paths funnelling generated charges upon localised states [21]. 

**Figure 4:**
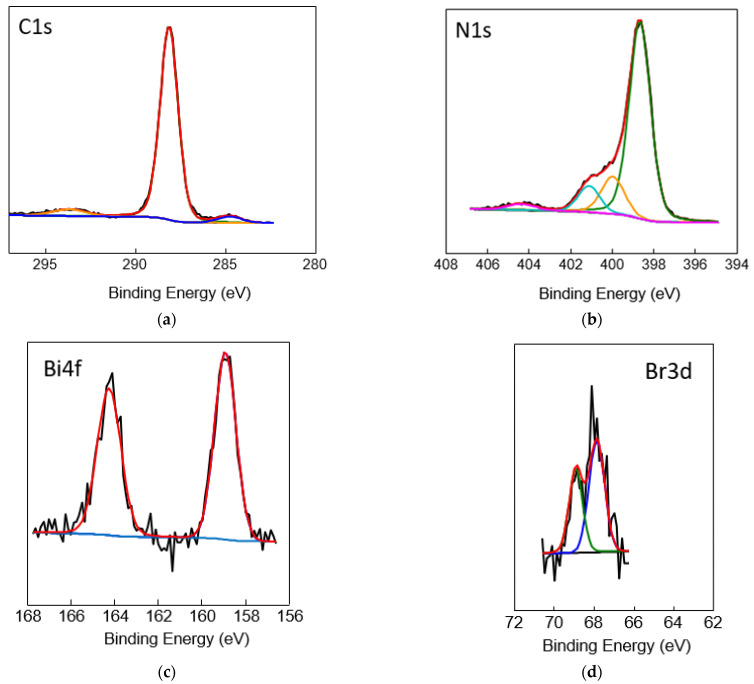
XPS analysis of the 0.02 wt.% Cs_3_Bi_2_Br_9_ NCs/g-C_3_N_4_ showing the high-resolution spectra of the (**a**) C 1s, (**b**) N 1s, (**c**) Br 3d and (**d**) Bi 4f. The black line corresponds to the obtained spectrum, the red line to the envelope-fitting curve and the remaining-coloured lines correspond to the fitted peaks.

The surface chemical composition was investigated via XPS analysis (Figure 4) of a representative sample (0.02 wt.%Cs_3_Bi_2_Br_9_/g-C_3_N_4_ NSs). Figure S7 shows the survey spectra of the composite. Figure 4a shows the high-resolution spectrum of the C 1s. The spectrum is dominated by the characteristic peak at 288.2 eV originating from the sp^2^ C atom connected to a N atom (N–C=N) from the aromatic ring of g-C_3_N_4_. The peak at 284.8 eV and 293.7 eV corresponds to C–C bonds (3.8% of the total C 1s) and the π-π* transition, respectively, with the latter being typical of aromatic carbon compounds. Figure 4b shows the high-resolution N 1s, the peak at 398 eV can be attributed to the sp^2^ bond N atom in the triazine ring (–C=N–), while the presence of a low-intensity shoulder peak at 399.9 eV originated from the bridging nitrogen atom with tertiary C atoms, N–(C)3. The peak at 401.1 eV can be indicative of –NH_x_ groups. The weak peak at 404.3 eV can be attributed to oxidised species because g-C_3_N_4_ NSs are obtained by the thermal exfoliation of bulk g-C_3_N_4_ in air. Figure 4c,d show the separation of the spin-orbital components for the Bi (4f_7/2_, 4f_5/2_) and Br (3d_5/2_, 3d_3/2_), the values were 5.32 eV and 1.05 eV, which indicate the oxidation state of Bi and Br are +3 and −1, respectively. Figure S8 shows the high-resolution spectrum of the Cs 3d analysis. These results confirm the presence of the Cs_3_Bi_2_Br_9_ in the composite with the smallest perovskite loading. 

**Figure 5:**
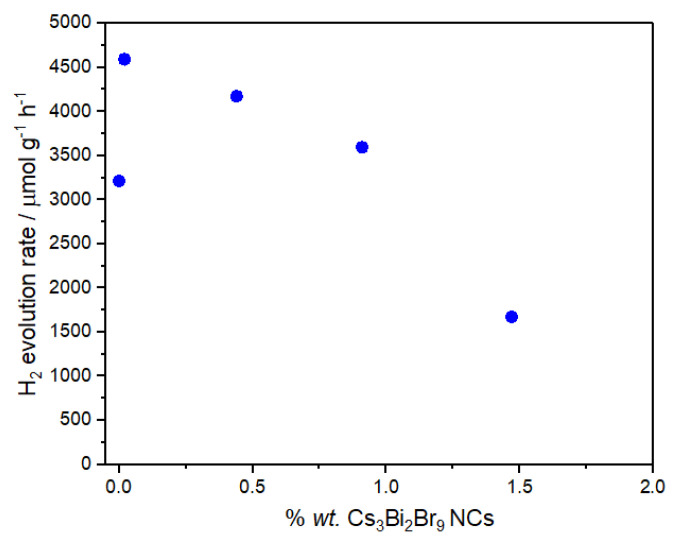
Hydrogen evolution reaction of the Cs_3_Bi_2_Br_9_ NCs/g-C_3_N_4_ NSs composites observed at different percentages of perovskite loading (wt.%); conditions: 10 v/v %TEOA aqueous solution, 1 g L^−1^ catalyst, 3 wt.%Pt as co-catalyst, simulated solar light (6 h, 500 W m^−2^); RSD < 10% (*n* = 3).

The solar-driven photocatalytic efficiency of the prepared composites was determined in terms of hydrogen evolution reaction (HER) under standard test conditions, viz. 10% v/v TEOA aqueous solution, as a sacrificial agent and 3 wt.% platinum as co-catalyst. Figure 5 shows the HER results as a function of perovskite loading. HER of the g-C_3_N_4_ NSs achieved a value of 3 212 μmol g^−1^ h^−1^, which is most probably related to the high surface area achieved by thermal exfoliation of bulk g-C_3_N_4_. The incorporation of Cs_3_Bi_2_Br_9_ NCs into the g-C_3_N_4_ NSs resulted in a positive increase in the hydrogen photogeneration with mean values of 4 593, 4 173 and 3 595 (*n* = 3) when the loading of the perovskite in the composite was 0.02, 0.44 and 0.91 wt.%*.,* respectively. These results clearly indicate a synergic effect between the two semiconductors (Cs_3_Bi_2_Br_9_ NCs and g-C_3_N_4_ NSs). However, higher perovskite loadings resulted in a detrimental effect to HER, with the composite prepared at the highest perovskite loading (1.47 wt.%) producing just 1 674 μmol g^−1^ h^−1^. 

**Figure 6:**
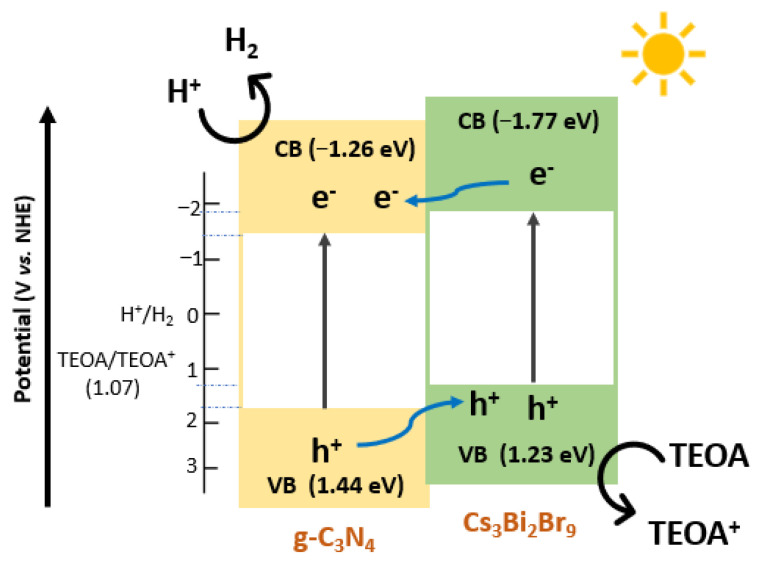
Diagram of charge transfer in the Cs_3_Bi_2_Br_9_ NCs/g-C_3_N_4_ NSs heterojunction for the photocatalytic H_2_ production under solar radiation.

As mentioned in the introduction, we previously investigated the analogous heterojunction made of microcrystalline Cs_3_Bi_2_Br_9_ and bulk g-C_3_N_4_ (not exfoliated), reporting a HER of 81 μmol g^−1^ h^−1^ for bulk carbon nitride, 22 μmol g^−1^ h^−1^ for the pure microcrystalline perovskite and the highest H_2_ production around 1 050 μmol g^−1^ h^−1^ achieved for the 2.5 wt.% bulk Cs_3_Bi_2_Br_9_ /bulk g-C_3_N_4_ composite, accompanied by a decrease of the HER when the loading of the perovskite exceeded 2.5 wt.% [21]. A similar trend with a synergistic effect between the two semiconductors is also observed here but with a shift towards higher hydrogen photogeneration at lower perovskite amounts, indicating a more efficient charge transfer at the interface between Cs_3_Bi_2_Br_9_ and g-C_3_N_4_ when nanostructured semiconductors are employed in the construction of the heterojunction. Based on available electronic structure data, the proposed mechanism of the actual band alignment between Cs_3_Bi_2_Br_9_ and g-C_3_N_4_ in the present heterojunction (Type II) is shown in Figure 6 [27,28,29]. 

Such an alignment favours efficient charge separation leading to the migration of the photogenerated holes from g-C_3_N_4_ NSs to Cs_3_Bi_2_Br_9_ NCs and, in turn, of the photogenerated electrons from Cs_3_Bi_2_Br_9_ NCs to g-C_3_N_4_ NSs resulting in an improvement of the HER in the heterojunction. The trend of the HER with the perovskite loading can be explained based on the self-trapping phenomena occurring in Cs_3_Bi_2_Br_9_ at higher concentrations because of polaron formation which seems to be an inactive mechanism at lower amounts of perovskite [21].

Finally, Table 1 lists the series of lead-free MHPs-based heterojunctions with g-C_3_N_4_ reported to date in the current literature for the photogeneration of hydrogen. 

## 4. Conclusions

In the present work, we synthesised Cs_3_B_i2_Br_9_ nanocrystals by a LARP method coupled with a planetary milling process. The Cs_3_B_i2_Br_9_ NCs were coupled to g-C_3_N_4_ nanosheets prepared by a simple and scalable thermal exfoliation method. A wet chemistry approach was employed to produce heterojunctions for hydrogen gas evolution under solar simulated light. The synergic effect achieved by combining both nanomaterials resulted in a H_2_ production of 4 593 μmol g^−1^ h^−1^ in correspondence with low loadings of Cs_3_Bi_2_Br_9_ NCs in the composite. In conclusion, this work provides further evidence of lead-free MHPs as highly active materials for photocatalytic applications, with considerable potential in the clean energy research field. 

## Figures and Tables

**Table 1:** HER values reported in the literature using lead-free perovskites/g-C_3_N_4_ composites.

Composite	HER/ µmol g^−1^ h^−1^	Experiment Conditions	Ref
Bulk Cs_3_Bi_2_Br_9_/bulk g-C_3_N_4_	1050	1 g_catalyst_/L_sol_, 10% *v/v* TEOA, 3 wt.%. Pt, 500 W m^−2^	[21]
Bulk PEA_2_SnBr_4_/bulk g-C_3_N_4_	1600	1 g_catalyst_/L_sol_, 10% *v/v* TEOA, 3 wt.% Pt, 500 W m^−2^	[30]
Bulk PhBz_2_Ge_2_Br_4_/bulk g-C_3_N_4_	1200	1 g_catalyst_/L_sol_, 10% *v/v* TEOA, 3 wt.%. Pt, 500 W m^−2^	[7]
Bulk Cs_3_Bi_2_I_9_/bulk g-C_3_N_4_	920	1 g_catalyst_/L_sol,_ 10% MeOH, 1 wt.% Pt, UV light irradiation at 355 nm.	[31]
Bulk DMASnBr_3_/bulk g-C_3_N_4_	1730	1 g_catalyst_/L_sol_, 10% *v/v* TEOA, 3 wt.% Pt, 500 W m^−2^	[32]
Cs_3_Bi_2_Br_9_ NCs/g-C_3_N_4_ NSs	4593	1 g_catalyst_/L_sol_, 10% *v/v* TEOA, 3 wt.% Pt, 500 W m^−2^	This work

Note: PEA, phenylethylammonium; PhBz, phenylbenzilammonium; DMA, dimethylammonium; NCs, nanocrystals; NSs, nanosheets; TEOA, triethanolamine; MeOH, Methanol.

## Data Availability

Not applicable.

## Supplementary Materials

The following supporting information can be downloaded at: https://www.mdpi.com/article/10.3390/nano13020263/s1, Figure S1: XRD pattern of the bulk g-C_3_N_4_ and the g- C_3_N_4_ NSs; Figure S2: Nitrogen adsorption isotherms of bulk g-C_3_N_4_ and g-C_3_N_4_ nanosheets. Figure S3: SEM micrograph of the bulk g-C_3_N_4_; Figure S4: EDX analysis on two heterojunctions. Figure S5: BSE-HRSEM micrograph of the 0.44 wt.% Cs_3_Bi_2_Br_9_ NCs/g-C_3_N_4_ NSs. Figure S6: (a) Enlarge section of the Tauc plot and (b) absorbance spectra versus wavelength. Figure S7: Survey spectra of the 0.02 wt.% Cs_3_Bi_2_Br_9_ /g-C_3_N_4_ NSs. Figure S8: High-resolution XPS analysis of Cs 3d for the 0.02 wt.% Cs_3_Bi_2_Br_9_/g-C_3_N_4_ NSs.

## Abbreviations

metal halide perovskites (MHP), nanosheets (NSs), hydrogen evolution rate (HER), nanocrystals (NCs), wet ball milling (WBM), ligand-assisted reprecipitation (LARP).
