# Supplementary material for: Preparation of Heterojunctions Based on Cs3Bi2Br9 Nanocrystals and g-C3N4 Nanosheets for Photocatalytic Hydrogen Evolution
Source: Nanomaterials (Basel). 2023 Jan 7;13(2):263. doi: 10.3390/nano13020263 (PMC9866070; doi:10.3390/nano13020263)
Supplement: Supplementary file 1 [file nanomaterials-13-00263-s001.zip › nanomaterials-2087237-supplementary.pdf]

Supporting information

Preparation of Heterojunctions Based on $Cs_3Bi_2Br_9$ Nanocrystals and g-C_3N_4 Nanosheets for Photocatalytic Hydrogen Evolution

María Medina-Llamas [1,2,*], Andrea Speltini [2], Antonella Profumo [2], Francesca Panzarea [3], Antonella Milella [3], Francesco Fracassi [3,4], Andrea Listorti [3] and Lorenzo Malavasi [2,*]

1 Unidad Académica Preparatoria, Plantel II, Universidad Autónoma de Zacatecas, Zacatecas 98068, México
2 Department of Chemistry, University of Pavia, Via Taramelli 12, 27100 Pavia, Italy
3 Department of Chemistry, University of Bari "Aldo Moro" Via Orabona 4, 70126 Bari, Italy
4 National Research Council, Department of Chemistry, Institute of Nanotechnology (CNR-NANOTEC), 70125 Bari, Italy
* Correspondence: maria.medina@uaz.edu.mx (M.M.-L.); lorenzo.malavasi@unipv.it (L.M.)

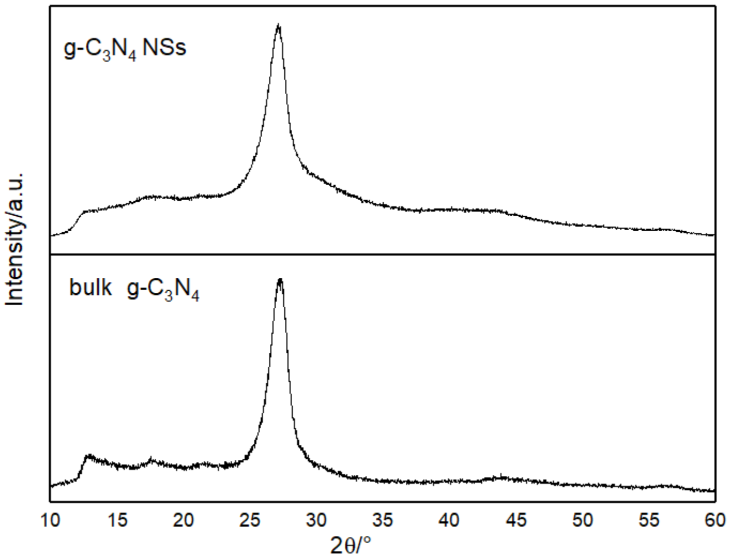

Figure S1. XRD pattern of the bulk g-C_3N_4 and the g-C_3N_4 nanosheets.

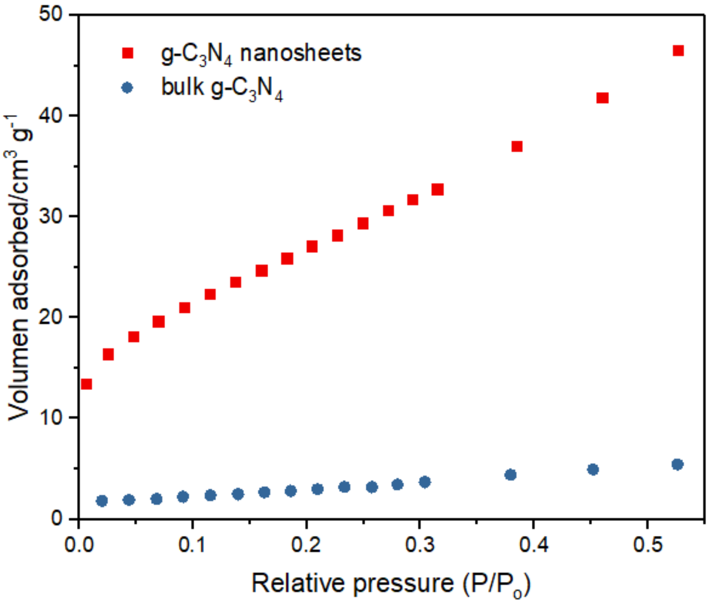

Figure S2 Nitrogen adsorption isotherms of bulk g-C_3N_4 and g-C_3N_4 nanosheets.

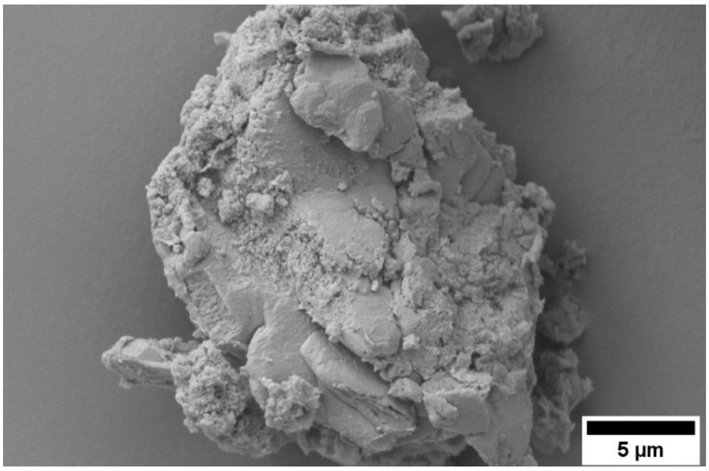

Figure S3. SEM micrograph of the bulk g-C_3N_4.

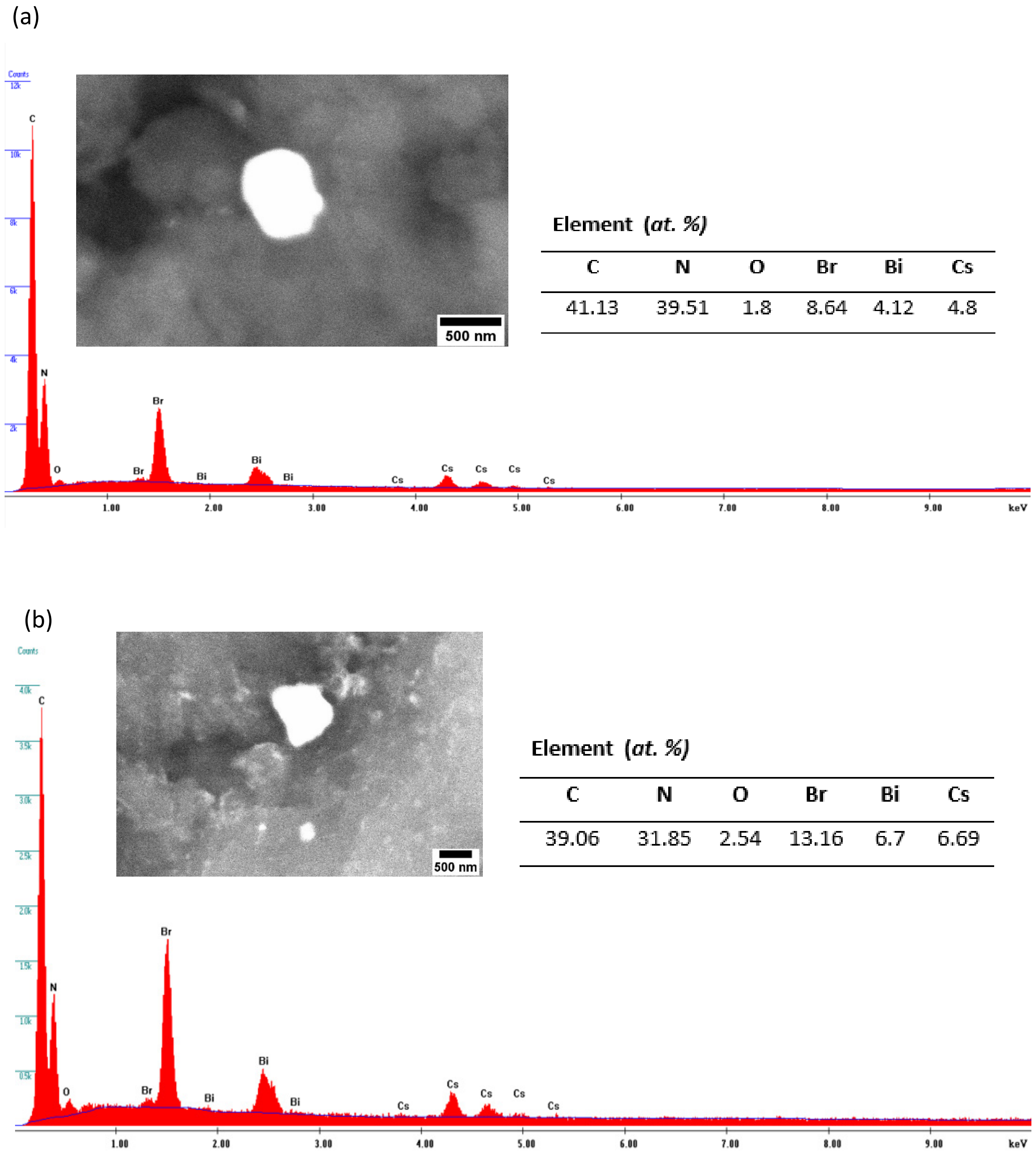

(a) Element (*at. %*)

C	N	O	Br	Bi	Cs
41.13	39.51	1.8	8.64	4.12	4.8

(b) Element (*at. %*)

C	N	O	Br	Bi	Cs
39.06	31.85	2.54	13.16	6.7	6.69

Figure S4. EDX analysis on the heterojunctions (**a**) 0.44wt. % $Cs_3Bi_2Br_9$ NCs/g-C_3N_4 NSs and the (**b**) 1.47 wt. % $Cs_3Bi_2Br_9$ NCs/g-C_3N_4 NSs.

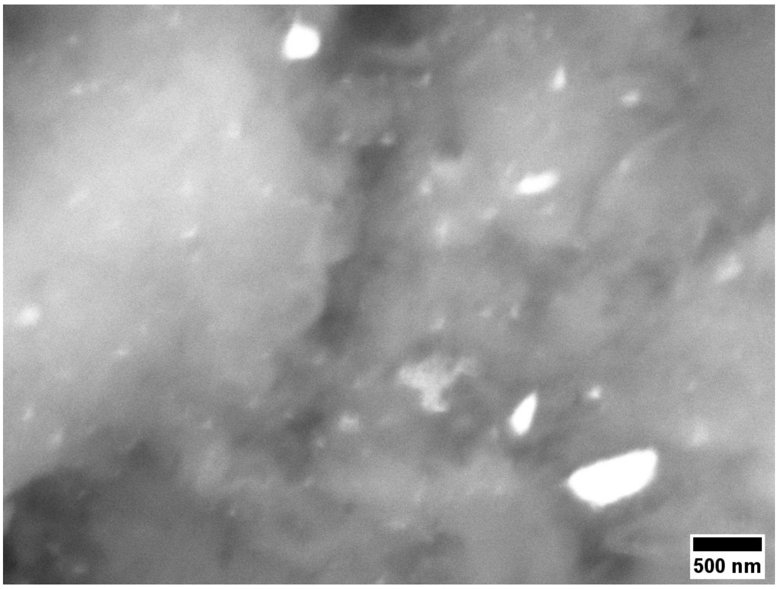

Figure S5. BSE-HRSEM micrograph of the 0.44 wt. % $Cs_3Bi_2Br_9$ NCs/g-C_3N_4 NS.

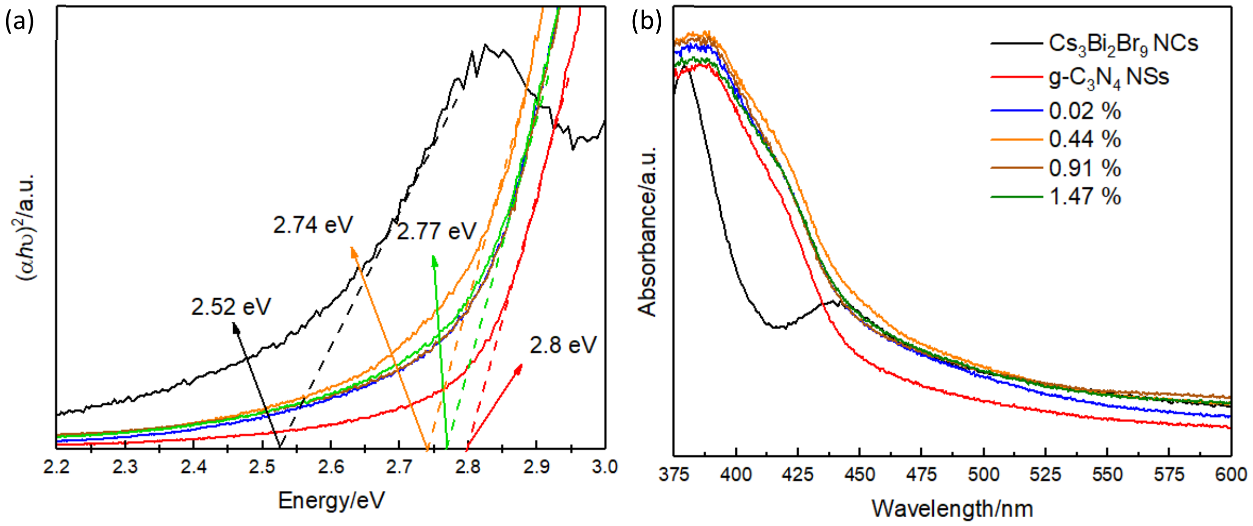

Figure S6. (**a**) Enlarge section of the Tauc plot and (**b**) absorbance spectra versus wavelength of the g-C_3N_4 NSs, the $Cs_3Bi_2Br_9$ NCs and the $Cs_3Bi_2Br_9$/g-C_3N_4 NSs composites produced at different percentages of perovskite loading (wt. %).

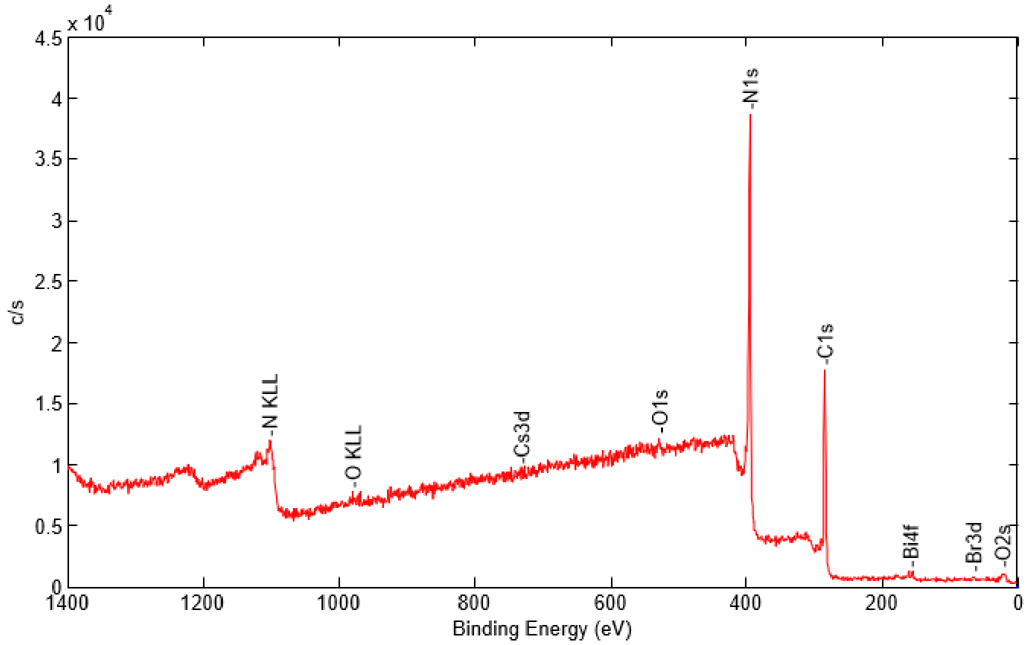

Figure S7. Survey spectra of the 0.02 wt. % $Cs_3Bi_2Br_9$/g-C_3N_4 NSs.

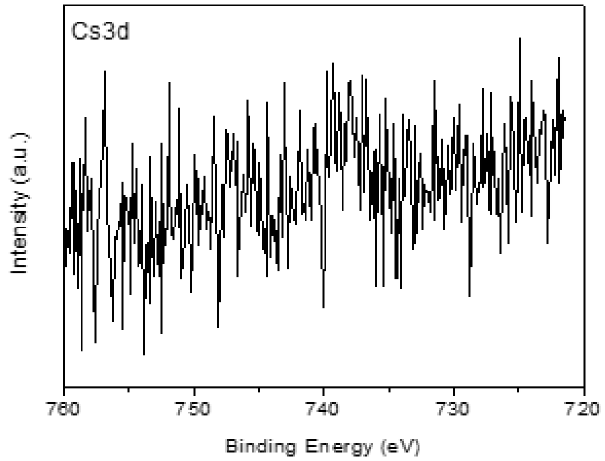

Figure S8. High-resolution XPS analysis of Cs 3*d* for the 0.02 wt. % $Cs_3Bi_2Br_9$/g-C_3N_4 NSs.
